# Interactions among Obstructive Sleep Apnea Syndrome Severity, Sex, and Obesity on Circulatory Inflammatory Biomarkers in Patients with Suspected Obstructive Sleep Apnea Syndrome: A Retrospective, Cross-Sectional Study

**DOI:** 10.3390/ijerph17134701

**Published:** 2020-06-30

**Authors:** Ming-Feng Wu, Yu-Hsuan Chen, Hui-Chen Chen, Wei-Chang Huang

**Affiliations:** 1 Division of Chest Medicine, Department of Internal Medicine, Taichung Veterans General Hospital, Taichung 407, Taiwan; heriknoha@vghtc.gov.tw (M.-F.W.); huchen0328@gmail.com (H.-C.C.); 2 Department of Medical Laboratory Science and Biotechnology, Central Taiwan University of Science and Technology, Taichung 406, Taiwan; yhchen2@ctust.edu.tw; 3 Department of Life Sciences, National Chung-Hsing University, Taichung 402, Taiwan; 4 Department of Medical Technology, Jen-Teh Junior College of Medicine, Nursing and Management, Miaoli 350, Taiwan; 5 Department of Industrial Engineering and Enterprise Information, Tunghai University, Taichung 407, Taiwan

**Keywords:** cardiovascular risk, CRP, IL-6, interaction, obesity, obstructive sleep apnea syndrome severity, sex, TNF-α

## Abstract

The interaction among obstructive sleep apnea syndrome (OSAS) severity, sex, and obesity on cardiovascular risk as determined by serum levels of C-reactive protein (CRP), interleukin-6 (IL-6), and tumor necrosis factor-α (TNF-α) remains unclear. Therefore, this study aimed to analyze individual associations between these three OSAS characteristics and three cardiovascular biomarkers and to determine whether the relationship was affected by other features in patients with suspected OSAS. For all participants (*n* = 100), OSAS severity and sex had an interaction effect on IL-6 level (*p* = 0.030). Specifically, the male patients (*p* = 0.005) with severe OSAS had higher IL-6 levels than those with normal to moderate OSAS, but this relationship was not significant in the female patients (*p* = 0.438). Moreover, in patients with normal to moderate OSAS (*p* = 0.004), but not in those with severe OSAS (*p* = 0.824), the female patients had higher IL-6 levels than the male patients. Both CRP (*p* = 0.001) and IL-6 (*p* = 0.000) levels were higher in the obese group than in the non-obese group independently of OSAS severity and sex. The three features had no effects on TNF-α level individually and interactively. Our findings suggest that circulatory inflammatory markers should be comprehensively evaluated in this population and that treatment and preventive therapies should be modified accordingly.

## 1. Introduction

Obstructive sleep apnea syndrome (OSAS) is a disorder characterized by intermittent hypoxia during sleep followed by adipose tissue inflammation and has been shown to contribute to cardiovascular morbidity and mortality [1,2]. OSAS affects approximately 3% to 7% of adult men and 2% to 5% of adult women in the general population [3]. Full-night polysomnography (PSG) involves an overnight stay in a sleep laboratory with multichannel monitoring for sleep physiology and architecture, brain activity, and respiration during sleep and is the standard method used to diagnose and grade OSAS [4]. The primary treatment options include behavior modifications, continuous positive airway pressure (CPAP) devices, oral appliances, and surgery, which should be offered based on the severity of OSAS and the patient’s upper airway anatomy, preferences, and risk factors [5,6].

In addition to diverse patterns of craniofacial anomalies, the incidence and severity of OSAS are closely related to sex and body mass index (BMI), with male sex and a higher BMI associated with more severe OSAS compared to female sex and a lower BMI, respectively [7,8,9,10,11,12]. Accordingly, severe OSAS is more common in obese males. Furthermore, the severity of OSAS, sex, and BMI are all associated with the risk of cardiovascular disease, and male obese patients with more severe OSAS have been reported to have a higher incidence of cardiovascular morbidities than female non-obese patients with less severe OSAS [13,14,15,16,17]. Despite the apparent close relationship among these three characteristics (OSAS severity, sex, and obesity), little is known about their interaction effect on cardiovascular risk in such patients.

Inflammation is intimately involved in the development of atherosclerosis and the pathogenesis of cardiovascular disease and surveying inflammatory markers can help to identify individuals with increased inflammation who are at risk of future cardiovascular events. Several markers of inflammation are known predictors of cardiovascular risk, including C-reactive protein (CRP), interleukin-6 (IL-6), tumor necrosis factor-α (TNF-α), IL-8, and soluble intercellular adhesion molecule 1. Of these markers, CRP, IL-6 and TNF-α are the most frequently studied in overweight and OSAS patients and are thus the most representative [18,19,20,21].

We hypothesized that the three most common characteristics of OSAS (severity of OSAS, sex, and obesity) may have an interaction effect on the risk of cardiovascular events as determined by the serum levels of the three most representative inflammatory markers (CRP, IL-6, and TNF-α). Therefore, the aims of this study were to investigate the individual associations between the three features of OSAS and three markers of inflammation and to determine whether the association was affected by other features in patients with suspected OSAS. 

## 2. Materials and Methods 

### 2.1. Study Design, Setting, and Population

This retrospective, cross-sectional study adhered to the Strengthening the Reporting of Observational Studies in Epidemiology (STROBE) (Table S1) statement. It was conducted at Taichung Veterans General Hospital between May 2016 and April 2018. Patients were enrolled if they were aged ≥20 years, had not smoked for at least 1 year before enrollment, and had a suspected diagnosis of OSAS based on a sleep specialist’s comprehensive evaluation, including medical history, presenting symptoms and signs, physical examination, and Epworth Sleepiness Scale questionnaire, in a continuous unbiased sampling manner. Patients who had any infectious diseases, had taken any anti-inflammatory drugs within 1 month prior to enrollment, or had a history of any lung diseases such as asthma, chronic obstructive lung disease, lung cancer, bronchiectasis, and interstitial lung disease were excluded from the study. Patients who had a history of diabetes mellitus, chronic kidney disease, autoimmune disorders, or coronary artery disease and those who had participated in any investigational drug trial within 1 year were also excluded. The Institutional Review Board and Ethics Committee of Taichung Veterans General Hospital approved this study (approval number: CF11063). Written informed consent was obtained from all participating subjects.

### 2.2. Data Collection

Baseline information including demographics, waist circumference (WC), blood pressure (BP), plasma lipid profile measurements, and results of diagnostic PSG were collected for analysis. Body weight and height were measured in kilograms and centimeters, respectively, using a digital scale (KONGHO INSTRUMENT, HW-2020, New Taipei City, Taiwan) according to the manufacturer’s recommendations and BMI was calculated as

BMI = weight (kg)/height (m)^2^. (1)

BP was measured digitally using an upper arm BP monitor (Omron, HEM-7230, Kyoto, Japan) according to the manufacturer’s recommendations at the end of the PSG test. An inelastic tape with 1 mm precision was used to measure WC at the midpoint between the inferior costal margin and upper iliac crest [22].

### 2.3. Full-Night Polysomnography

The detailed methods of standard full-night PSG are available elsewhere [23]. In brief, all subjects who visited our “Snoring Clinics” and were suspected of having a diagnosis of OSAS underwent standard full-night PSG (Compumedics, E-series, Victoria, Australia). Four-channel electroencephalography, electrooculography, and submental and tibialis anterior electromyography were used to determine sleep stages and arousal index. Oro-nasal airflow, thermocouple, respiratory effort, and pulse oximetry were used to score apnea and hypopnea. The apnea-hypopnea index (AHI) was defined as the total number of apneas plus hypopneas divided by the total sleep time in hours. These events were manually scored based on the Task Force of the American Sleep Disorders Association, 2007 [24]. The severity of OSAS was graded as “normal” if the AHI was <5, “mild” if the AHI was ≥5 but <15, “moderate” if the AHI was ≥15 but <30, and “severe” for ≥30 events per hour [25].

### 2.4. Measurement of Blood Inflammatory Biomarker Levels

For the diagnostic PSG, all subjects went to bed at 10 pm and were naturally awakened between 5 am and 6 am. Venous blood samples were collected from all subjects between 5 am and 6 am just after awakening from PSG and were immediately put on ice. Serum was separated within 2 h of collection and samples were stored at −80 °C until analysis. Serum levels of CRP were measured using a latex particle-enhanced immunoturbidimetric assay [26]. Serum IL-6 and TNF-α levels were measured using an enzyme-linked immunosorbent assay (High Sensitivity Quantikine kit, R&D Systems, Minneapolis, MN) that could detect concentrations as low as 0.2 pg/mL and 0.5 pg/mL, respectively. Values were measured in duplicate and the average value was used for analysis.

### 2.5. Statistical Analysis

In this study, three bisected categorical predictors were required when performing the three-way interaction analysis. Therefore, the participants were categorized into three paired study groups based on the three features of interest (the severity of OSAS, sex, and obesity): normal to moderate OSAS group versus severe OSAS group, female group versus male group, and non-obese group versus obese group. Severe OSAS and obesity were defined as an AHI ≥ 30 events per hour and BMI ≥ 27 kg/m^2^, respectively, based on public health implications and different BMI cut-off points for obesity in different ethnic groups [13,27,28,29]. Otherwise, the patients were defined as having normal to moderate OSAS and being non-obese, respectively. All data were expressed as mean and standard deviation for continuous variables or a number (percentage) for categorical variables. Comparisons were made using the independent t-test for continuous variables based on the normality test where log_10_ transformation was performed when variables were non-parametric distribution and the chi-square test for categorical variables between the paired study groups. All of the available data were analyzed in cases where some data were missing. Three-way interaction analysis was used to determine the interaction effects of OSAS severity, sex, and obesity on the three studied inflammatory biomarkers (CRP, IL-6, and TNF-α). If there was no significant interaction among the three factors on the inflammatory biomarkers, then the simple main effect was analyzed between any two factors. In addition, the main effects were analyzed if there was no significance in the simple main effects. One-way analysis of variance (ANOVA) was used to validate differences if they were significant in the three-way interaction analysis. Post hoc statistical power was calculated using G-Power 3.1(Heinrich-Heine-Universität, Düsseldorf, Germany) for the significant findings using the criterion for significance (alpha) at 0.05. The statistical analyses were performed using Predictive Analysis Software (PASW) version 18.0 (SPSS Inc., Chicago, IL, USA). Significance was set at *p* < 0.05.

## 3. Results

**Figure 1:**
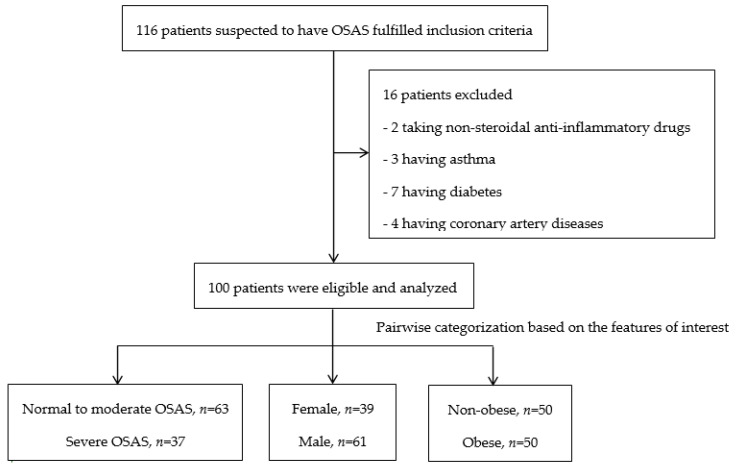
The patient enrollment flow chart. Abbreviations: OSAS, obstructive sleep apnea syndrome.

Of 151 patients screened, 116 (76.8%) patients fulfilled the inclusion criteria while 16 patients were excluded from this study thereafter. Thus, a total of 100 patients were included in the final analysis. Patient enrollment is detailed in Figure 1.

Table 1 and Table 2 show the demographic and PSG results of the enrolled participants. The mean ± standard deviation age of all participants was 45.5 ± 9.8 years. More than half of the participants were male (61/100, 61%) and had normal to moderate OSAS (63/100, 63%) and half of the participants were obese (50/100, 50%). The systolic (144.6 ± 19.4 mmHg vs. 130.9 ± 14.3 mmHg, *p* < 0.05) and diastolic (92.4 ± 16.3 mmHg vs. 81.9 ± 10.9 mmHg, *p* < 0.05) BPs and WC (103.0 ± 10.0 cm vs. 85.9 ± 8.5 cm, *p* < 0.05) were significantly higher in the obese group compared to the non-obese group, while the lowest oxygen saturation during sleep was higher in the participants with normal to moderate OSAS compared to those with severe OSAS (85.3 ± 6.4% vs. 74.4 ± 9.9%, *p* < 0.05). With regards to the studied inflammatory biomarkers, the obese patients had higher CRP (0.36 ± 0.35 mg/dL vs. 0.163 ± 0.29 mg/dL, *p* < 0.05) and IL-6 (1.877 ± 1.21 pg/mL vs. 0.978 ± 0.59 pg/mL, *p* < 0.05) serum levels than the non-obese patients, while the patients with severe OSAS had a higher IL-6 level than those with normal to moderate OSAS (1.787 ± 1.32 pg/mL vs. 1.216 ± 0.79 pg/mL, *p* < 0.05). Serum TNF-α levels were similar between all paired study groups (Table 3). The levels of the studied circulatory inflammatory biomarkers were obviously different among a variety of combinations of the three characteristics of interest (Table 4). This implied that there were complex relationships among the three features on the levels of blood inflammatory biomarkers.

The results of the three-way interaction analysis showed that there were no interaction effects among OSAS severity, sex, and obesity on any studied inflammatory biomarker (*p* = 0.494, *p* = 0.120, and *p* = 0.082 for CRP, IL-6, and TNF-α, respectively) (Table 5).

Further analysis of the interaction effect between any two of the three studied factors showed that the severity of OSAS and sex had a simple main effect on IL-6 level (*p* = 0.030) (Table 5). Cell means with the post hoc test showed that the IL-6 level was significantly higher in the severe OSAS group compared to that in the normal to moderate OSAS group in male (*p* = 0.005, statistical power = 83%) but not in female (*p* = 0.438) patients. In addition, in the patients with normal to moderate OSAS, the female patients had a significantly higher IL-6 level compared to the male patients (*p* = 0.004, statistical power = 88%) (Table 6). That is, an interaction between OSAS severity and sex on IL-6 levels was found.

As there was no interaction between obesity and OSAS severity or between obesity and sex, we performed further analysis which revealed a significant main effect of obesity on both CRP (*p* = 0.001) and IL-6 (*p* = 0.000) levels (Table 5). Post hoc analysis showed that both CRP (*p* = 0.001, statistical power = 92%) and IL-6 (*p* = 0.000, statistical power = 99%) levels were higher in the obese group than in the non-obese group (Table 6). In other words, obesity was associated with the levels of CRP and IL-6 independently of OSAS severity and sex.

**Figure 2:**
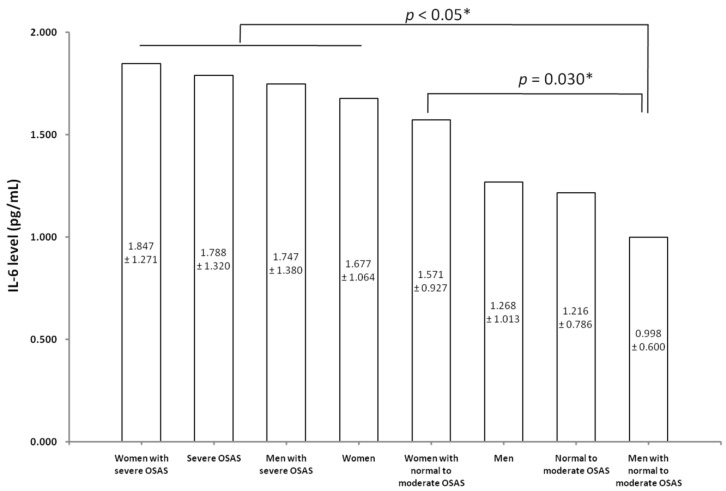
The validation results of the interaction effects between OSAS severity and sex on the level of IL-6. All data are expressed as mean and standard deviation at a significant difference with a *p*-value <0.05 (*). Abbreviations: IL-6, interleukin-6; OSAS, obstructive sleep apnea syndrome.

One-way ANOVA was used to validate the findings of the interaction effect between OSAS severity and sex on IL-6 levels (Figure 2). Female patients with severe OSAS had the highest serum level of IL-6, while male patients with normal to moderate OSAS had the lowest level of IL-6. These results were consistent with those shown in Table 6.

The three studied factors had no effects on TNF-α level individually and interactively in the three-way interaction analysis.

## 4. Discussion

### 4.1. Main Findings

There was no significant interaction among the three features on the studied inflammatory biomarkers. Nevertheless, this study showed that the relationship between the severity of OSAS and IL-6 levels was altered by sex and vice versa in patients with suspected OSAS. In addition, obesity was independently associated with both serum CRP and IL-6 levels without interacting with OSAS severity and sex, while the three features had no effect on TNF-α level individually and interactively.

### 4.2. Interpretation of the Findings in Relation to Previously Published Work

Arnardottir et al. and Kim et al. reported an interaction effect between OSAS severity and BMI on both IL-6 and/or CRP levels and suggested that the degree of obesity altered the relationships between OSAS severity and IL-6 concentrations and/or between OSAS severity and CRP levels [30,31]. In contrast, we found that OSAS severity and sex had an interaction effect on IL-6 level. Specifically, the patients with severe OSAS had a significantly higher level of IL-6 than those with normal to moderate OSAS, although the relationship was only found in men. Moreover, women had a significantly higher IL-6 level compared to men, although this association was only found in patients with normal to moderate OSAS. The difference between these three studies could be due to the different study designs. In the current study, we enrolled all patients with OSAS ranging from normal to severe and excluded current smokers and subjects with lung diseases, diabetes mellitus, and coronary artery disease. In comparison, Arnardottir et al. only included subjects with moderate to severe OSAS with an AHI > 15 events/hour and those with factors that may have confounded the serum levels of inflammatory biomarkers, including current smokers and those with cardiovascular diseases, diabetes, and chronic obstructive pulmonary disease. Similar to Arnardottir et al.’s study, Kim et al. also enrolled participants with characteristics that may have had an impact on circulatory inflammatory biomarkers, although they included patients with a broader range of OSAS severity, from normal to severe. Furthermore, almost 40% of our participants were female compared to 12.3% and 47.2% in Arnardottir et al.’s study and Kim et al.’s study, respectively [30,31]. Taken together, this suggests that the characteristics of OSAS that are responsible for the interaction effect on inflammatory markers vary according to the studied sub-population with OSAS.

Consistent with our results that obesity was associated with elevated serum levels of CRP and IL-6 independently of OSAS severity and sex, several studies have also reported increases in both CRP and IL-6 levels in overweight and obese patients regardless of sex [21,32,33]. This may be because IL-6 is secreted by adipose tissue [34]. Moreover, CRP is produced in the liver following IL-6 stimulation so that patients with a higher BMI may be more likely to have a higher level of IL-6 and its hepatic product, CRP. These findings indicate that independent of sex, obesity represents an inflammatory state involving the pathogenesis and progression of atherosclerosis and coronary artery disease and that the presence of obesity is associated with a higher risk of cardiovascular disease [35,36,37,38,39,40,41].

In contrast to our findings that none of the features of interest (OSAS severity, sex, and obesity) were associated with serum TNF-α level, Vgontzas et al. reported significant increases in TNF-α level in patients with OSAS compared to normal subjects [42]. This may be due to the different characteristics of the controls used for comparisons between the two studies. The baseline data, and especially age, were more evenly balanced between the paired study groups in our study, whereas Vgontzas et al. compared the plasma level of TNF-α between middle-aged OSAS patients and younger normal controls (mean age: 40.9 ± 2.2, mean age: 24.1 ± 0.8 years, respectively) [42].

Circulating cardiac biomarkers, and in particular, N-terminal brain natriuretic peptide (NT-proBNP) and high-sensitive troponin T (hs-TropT), are potential indicators of myocardial stretch and predictors of clinical outcomes in patients with heart failure and coronary artery disease [39,43]. However, evidence is conflicting on the associations between AHI and the level of NT-proBNP and between AHI and the level of hs-TropT in patients suspected of having OSAS. Previous studies have not found a correlation between AHI and the level of NT-proBNP after adjusting for potential confounding factors, while Ljunggren et al. reported a dose–response relationship between the severity of OSA and NT-proBNP levels in female patients [44,45,46]. Moreover, several other studies reported that the severity of OSAS was not independently associated with the level of hs-TropT, although the hs-TropT level was correlated with AHI in patients with severe OSAS and coexisting coronary artery disease [47,48]. Therefore, future studies are needed to investigate the feasibility of these two cardiac biomarkers as predictors of cardiovascular risk in patients with suspected OSAS.

### 4.3. Strengths and Limitations of this Study

The strengths of this study include the fact that subjects with any confounding factors for the levels of inflammatory biomarkers were excluded. Furthermore, the venous blood collected for the measurement of the studied inflammatory biomarkers was drawn after natural awakening from PSG without constraints that may have influenced the level of blood inflammatory biomarkers. Therefore, our findings represent the true interaction effect of the three studied features on the blood inflammatory biomarkers of interest. This compensates for several limitations, including the fact that the ethnic-specific BMI cut-off value of 27 kg/m^2^ was used to define obesity in this Asian population, and data on menstrual cycle and menopause in the female participants, co-morbidities, craniofacial morphology, arterial carbon dioxide levels, and other potential cardiac biomarkers were not recorded and/or analyzed because of the study design. Moreover, the PSG cables may have impacted the sleep architecture and thereby the AHI and levels of the studied circulatory inflammatory biomarkers. This means that bias due to ethnicity and its corresponding BMI cut-off point for obesity, hormonal effects, pulmonary and extra-pulmonary inflammatory disorders, upper airway anomalies, and technical limitations on the serum levels of the studied inflammatory biomarkers may have existed. Furthermore, relatively few circulating inflammatory markers were explored and the presence of obesity hypoventilation syndrome was not investigated in this study.

### 4.4. Implications for Future Research, Policy, and Practice

Our findings indicate that male patients with severe OSAS, female patients with normal to moderate OSAS, and obese patients with OSAS have higher levels of systemic inflammation associated with the pathogenesis and progression of cardiovascular diseases compared to male patients with normal to moderate OSAS, male patients with normal to moderate OSAS, and non-obese patients with OSAS, respectively. Our findings highlight that different characteristics of patients with suspected OSAS have an interaction effect on serum levels of circulating cardiac biomarkers. Taken together with evidence that nasal CPAP rather than mandibular advancement devices can decrease the levels of inflammatory markers [21,49], our study indicates the importance of comprehensively evaluating levels of inflammatory markers that may affect treatment and preventive therapies when managing patients with suspected OSAS. Future well-designed studies should enroll a larger, multicultural cohort with detailed information about menstrual cycle and menopause for the female participants, co-morbidities, upper airway anatomical characteristics, and arterial carbon dioxide values and evaluate more cardio-specific biomarkers to validate our results.

## 5. Conclusions

We found an interaction effect between the severity of OSAS and sex on serum IL-6 levels. Our findings suggest that clinicians should comprehensively evaluate circulating cardiac markers of inflammation and then adjust the therapeutic and preventive strategies accordingly when managing patients with suspected OSAS.

## Figures and Tables

**Table 1:** Baseline demographics of the enrolled participants.

Characteristic	All (*n* = 100)	Normal to Moderate OSAS (*n* = 63)	Severe OSAS (*n* = 37)	Female (*n* = 39)	Male (*n* = 61)	Non-Obese (*n* = 50)	Obese (*n* = 50)
Sex							
Male	61 (61%)	39 (61.9%)	22 (59.5%)	0 (0%)	61 (100%)	29 (58%)	32 (64%)
Female	39 (39%)	24 (38.1%)	15 (40.5%)	39 (100%)	0 (0%)	21 (42%)	18 (36%)
Age (years)	45.5 ± 9.8	45.2 ± 8.7	46.3 ± 11.5	47.4 ± 7.7	44.3 ± 10.9	46.5 ± 9.4	44.5 ± 10.2
WC (cm) ^a^	94.5 ± 12.6	93.3 ± 13.3	96.4 ± 11.3	91.6 ± 14.5	96.3 ± 11.0	85.9 ± 8.5	103.0 ± 10.0
SBP (mmHg)	136.8 ± 17.8	135.4 ± 20.1	138.6 ± 14.6	134.8 ± 16.4	137.9 ± 18.8	130.9 ± 14.3	144.6 ± 19.4
DBP (mmHg)	86.4 ± 14.4	84.9 ± 13.7	88.3 ± 15.1	83.8 ± 13.9	87.8 ± 14.6	81.9 ± 10.9	92.4 ± 16.3
BMI (kg/m^2^) ^a^	27.8 ± 5.3	27.4 ± 5.4	28.4 ± 5.1	28.1 ± 6.7	27.6 ± 4.2	23.7 ± 2.2	31.9 ± 4.3
≥27	50 (50%)	31 (49.2%)	19 (51.4%)	18 (46.2%)	32 (52.5%)	0 (0%)	50 (100%)
<27	50 (50%)	32 (51.4%)	18 (48.6%)	21 (53.8%)	29 (47.5%)	50 (100%)	0 (0%)

^a^*p* < 0.05 between the non-obese and obese groups. Abbreviations: BMI, body mass index; DBP, diastolic blood pressure; OSAS, obstructive sleep apnea syndrome; SBP, systolic blood pressure; WC, waist circumference.

**Table 2:** Polysomnography findings of all participants.

Parameter	All (*n* = 100)	Normal to Moderate OSAS (*n* = 63)	Severe OSAS (*n* = 37)	Female (*n* = 39)	Male (*n* = 61)	Non-Obese (*n* = 50)	Obese (*n* = 50)
AHI (events/hour) ^b^	23.9 ± 23.5	8.9 ± 8.6	49.5 ± 18.1	24.2 ± 23.0	23.8 ± 24.1	20.7 ± 19.3	27.2 ± 26.9
>30	37 (37%)	0 (0%)	37 (100%)	15 (38.5%)	22 (36.1%)	18 (36%)	19 (38%)
≤30	63 (63%)	63 (100%)	0 (0%)	24 (61.5%)	39 (63.9%)	32 (64%)	31 (62%)
OAI (events/hour) ^κ,b^	8.9 ± 13.2	2.6 ± 4.3	19.4 ± 16.4	7.6 ± 10.0	9.7 ± 15.0	8.4 ± 10.0	9.3 ± 16.0
CAI (events/hour) ^κ,b^	1.3 ± 2.6	0.6 ± 1.9	2.5 ± 3.2	1.0 ± 1.9	1.5 ± 2.9	1.8 ± 3.3	0.8 ± 1.6
MAI (events/hour) ^κ,b,c^	1.5 ± 6.2	0.2 ± 0.7	3.8 ± 9.9	0.2 ± 0.4	2.4 ± 7.9	1.7 ± 7.7	1.3 ± 4.3
HI (events/hour) ^κ,a,b^	12.4 ± 14.1	5.7 ± 5.1	24.0 ± 16.8	15.5 ± 17.8	10.5 ± 10.8	8.8 ± 9.0	16.1 ± 17.1
Arousal index (events/hour) ^κ,b^	16.5 ± 18.3	7.4 ± 5.9	31.9 ± 21.7	15.4 ± 6.9	17.1 ± 19.2	14.5 ± 14.1	18.3 ± 21.6
Stages of sleep							
N1 (%) ^κ,a,b^	24.3 ± 14.3	20.6 ± 11.3	30.5 ± 16.7	23.2 ± 14.0	25.0 ± 14.6	21.0 ± 12.4	27.6 ± 15.5
N2 (%)	50.2 ± 12.9	50.5 ± 11.0	49.7 ± 15.4	48.7 ± 14.1	51.1 ± 11.9	50.7 ± 11.8	49.7 ± 13.8
N3 (%) ^b,c^	12.8 ± 8.9	15.2 ± 9.0	8.6 ± 7.1	15.5 ± 8.7	11.1 ± 8.7	14.2 ± 9.4	11.4 ± 8.3
R (%) ^κ^	12.7 ± 6.5	13.6 ± 6.0	11.1 ± 7.0	12.6 ± 6.9	12.7 ± 6.2	14.1 ± 5.8	11.4 ± 6.8
Sleep efficiency (%) ^κ^	86.4 ± 11.0	86.9 ± 10.6	85.5 ± 11.7	85.4 ± 13.0	87.0 ± 9.5	87.4 ± 10.1	85.4 ± 11.7
SpO_2nadir_ (%) ^κ,b^	81.3 ± 9.4	85.3 ± 6.4	74.4 ± 9.9	81.6 ± 10.2	81.1 ± 9.0	83.1 ± 8.2	79.5 ± 10.3
SpO_2mean_^a,b^	94.1 ± 4.0	95.7 ± 1.4	91.5 ± 5.4	94.2 ± 5.4	94.1 ± 2.8	94.9 ± 2.4	93.3 ± 5.0
TST (min) ^κ^	324.8 ± 44.0	328.0 ± 42.2	319.1 ± 47.0	320.4 ± 48.2	327.6 ± 41.2	331.7 ± 42.7	317.8 ± 44.5
ESS	10.2 ± 4.4	9.7 ± 4.5	11.1 ± 4.3	10.4 ± 4.6	10.0 ± 4.4	9.6 ± 4.3	10.8 ± 4.6

^a^*p* < 0.05 between the non-obese and obese groups. ^b^ *p* < 0.05 between the normal to moderate and severe OSAS groups. ^c^ *p* < 0.05 between the male and female groups. ^κ^ The comparison was based on log_10_ transformation. Abbreviations: AHI, apnea-hypopnea index; CAI, central apnea index; ESS, Epworth sleepiness scale; HI, hypopnea index; MAI, mixed apnea index; OAI, obstructive apnea index; OSAS, obstructive sleep apnea syndrome; R, rapid eye movement; SpO_2mean_, the average oxygen saturation during sleep; SpO_2nadir_, the lowest oxygen saturation during sleep.

**Table 3:** Blood measurements of the studied participants.

Variable	All (*n* = 100)	Normal to Moderate OSAS (*n* = 63)	Severe OSAS (*n* = 37)	Female ( *n* = 39)	Male (*n* = 61)	Non-Obese (*n* = 50)	Obese (*n* = 50)
WBC (/uL) ^κ,a,c^	7052.41 ± 2701.399	7152.22 ± 2941.925	6852.78 ± 2206.573	6180.83 ± 1641.815	7749.67 ± 3172.926	6089.17 ± 2190.213	7823.00 ± 2854.859
Cholesterol (mg/dL)	191.2 ± 37.3	187.9 ± 36.4	197.2 ± 38.5	194.6 ± 41.9	189.2 ± 34.2	192.1 ± 39.3	190.5 ± 35.5
Triglycerides(mg/dL)	145.2 ± 80.1	134.7 ± 70.3	163.0 ± 92.7	136.7 ± 76.6	150.6 ± 82.4	133.8 ± 80.2	156.6 ± 79.1
HDL (mg/dL) ^κ,a,c^	52.3 ± 12.0	51.2 ± 12.6	54.9 ± 10.2	58.6 ± 11.0	45.4 ±9.0	57.3 ± 12.0	48.0 ± 10.3
LDL (mg/dL)	119.5 ± 28.6	124.4 ± 28.3	110.2 ± 28.0	126.3 ± 31.3	114.0 ± 25.8	118.6 ± 33.8	120.4 ± 23.9
CRP (mg/dL) ^κ,a^	0.259 ± 0.33	0.227 ± 0.31	0.314 ± 0.37	0.319 ± 0.42	0.221 ± 0.26	0.163 ± 0.29	0.36 ± 0.35
IL-6 (pg/mL) ^κ,a,b,c^	1.428 ± 1.05	1.216 ± 0.79	1.787 ± 1.32	1.677 ± 1.06	1.268 ± 1.01	0.978 ± 0.59	1.877 ± 1.21
TNF-α (pg/mL)	1.160 ± 0.42	1.140 ± 0.41	1.194 ± 0.43	1.222 ± 0.41	1.120 ± 0.42	1.153 ± 0.44	1.167 ± 0.40

^a^*p* < 0.05 between the non-obese and obese groups. ^b^ *p* < 0.05 between the normal to moderate and severe OSAS groups. ^c^ *p* < 0.05 between the male and female groups. ^κ^ The comparison was based on log_10_ transformation. Abbreviations: CRP, C-reactive protein; HDL, high density lipoprotein; IL-6, interleukin-6; LDL, low density lipoprotein; OSAS, obstructive sleep apnea syndrome; TNF-α, tumor necrosis factor-α; WBC, white blood cell.

**Table 4:** The levels of circulatory inflammatory biomarkers in various combinations of the three studied features.

Features	CRP (mg/dL)	IL-6 (pg/mL)	TNF-α (pg/mL)
Normal to moderate OSAS, male and non-obese (*n* = 17)	0.142 ± 0.253	0.681 ± 0.259	0.915 ± 0.354
Normal to moderate OSAS, male and obese (*n* = 22)	0.224 ± 0.236	1.243 ± 0.676	1.238 ± 0.408
Normal to moderate OSAS, female and non-obese (*n* = 15)	0.182 ± 0.409	1.099 ± 0.638	1.237 ± 0.451
Normal to moderate OSAS, female and obese (*n* = 9)	0.467 ± 0.294	2.358 ± 0.801	1.166 ± 0.356
Severe OSAS, male and non-obese (*n* = 12)	0.152 ± 0.214	1.098 ± 0.553	1.308 ± 0.476
Severe OSAS, male and obese (*n* = 10)	0.430 ± 0.276	2.526 ± 1.684	0.967 ± 0.336
Severe OSAS, female and non-obese (*n* = 6)	0.191 ± 0.227	1.280 ± 0.920	1.307 ± 0.321
Severe OSAS, female and obese (*n* = 9)	0.483 ± 0.574	2.226 ± 1.377	1.196 ± 0.494

Abbreviations: CRP, C-reactive protein; IL-6, interleukin-6; OSAS, obstructive sleep apnea syndrome; TNF-α, tumor necrosis factor-α.

**Table 5:** Three-way interaction analysis by the studied inflammatory biomarkers.

Factor ^a^	CRP (*n* = 100) ^κ^					IL-6 (*n* = 100) ^κ^					TNF-α (*n* = 100)				
	SS	df	MS	F	*p*-value	SS	df	MS	F	*p*-value	SS	df	MS	F	*p*-value
A	0.814	1	0.814	2.126	0.148	0.208	1	0.208	4.123	0.045 *	0.065	1	0.065	0.389	0.534
B	0.613	1	0.613	1.601	0.209	0.285	1	0.285	5.639	0.020 *	0.303	1	0.303	1.809	0.182
C	7.620	1	7.620	19.913	0.001 *	1.839	1	1.839	36.370	0.000 *	0.053	1	0.053	0.317	0.575
A × B	0.128	1	0.128	0.335	0.564	0.347	1	0.347	6.869	0.010 *	0.001	1	0.001	0.004	0.951
A × C	0.154	1	0154	0.402	0.528	0.002	1	0.002	0.030	0.862	0.657	1	0.657	3.924	0.051
B × C	0.004	1	0.004	0.012	0.914	0.003	1	0.003	0.056	0.814	0.036	1	0.036	0.214	0.645
A × B × C	0.482	1	0.482	1.259	0.265	0.038	1	0.038	0.757	0.386	0.516	1	0.516	3.083	0.082
SSE	34.821	91	0.383			4.651	92	0.051			15.231	91	0.167		
SST	141.576	99				8.030	100				150.358	99			

* *p* <0.05. ^a^A, B, and C represent OSAS severity, sex, and obesity, respectively. ^κ^ The analysis was based on log_10_ transformation. Abbreviations: CRP, C-reactive protein; df, degree of freedom; F, F-value; IL-6, interleukin-6; MS, mean square; SS, total sum of square of deviation from mean; SSE, error sum of square; SST, total sum of square; TNF-α, tumor necrosis factor-α.

**Table 6:** Post hoc test (Scheffe) for the inflammatory biomarkers significantly associated with OSAS severity, sex, and obesity.

Variable	Factor	Three-Way Analysis	Post Hoc Test	Results	*p*-Value	Power
CRP	Obesity	Main effect	Marginal means	Obesity > Non-obesity	0.001 *	0.92
IL-6	Sex	Simple main effect	Cell means			0.88
	N-M OSAS			Female > Male	0.004 *	
	Severe OSAS			Female > Male	0.824	
IL-6	OSAS severity	Simple main effect	Cell means			
	Male			Severe OSAS > N-M OSAS	0.005 *	0.83
	Female			Severe OSAS > N-MOSAS	0.438	
IL-6	Obesity	Main effect	Marginal means	Obesity > Non-obesity	0.000 *	0.99

* *p* <0.05. Abbreviations: CRP, C-reactive protein; IL-6, interleukin-6; N-M, normal to moderate; OSAS, obstructive sleep apnea syndrome.

## Supplementary Materials

The following are available online at https://www.mdpi.com/1660-4601/17/13/4701/s1, Table S1: STROBE Statement—checklist of items that should be included in reports of observational studies.

## References

[1] Shahar E., Whitney C.W., Redline S., Lee E.T., Newman A.B., Nieto F.J., O’Connor G.T., Boland L.L., Schwartz J.E., Samet J.M. (2001). Sleep-disordered Breathing and Cardiovascular Disease. Am. J. Respir. Crit. Care Med..

[2] Yacoub M., Youssef I., O Salifu M., McFarlane S.I. (2018). Cardiovascular Disease Risk in Obstructive Sleep apnea: An Update. J. Sleep Disord. Ther..

[3] Punjabi N.M. (2008). The epidemiology of adult obstructive sleep apnea. Proc. Am. Thorac. Soc..

[4] Kapur V.K., Auckley D.H., Chowdhuri S., Kuhlmann D.C., Mehra R., Ramar K., Harrod C.G. (2017). Clinical Practice Guideline for Diagnostic Testing for Adult Obstructive Sleep Apnea: An American Academy of Sleep Medicine Clinical Practice Guideline. J. Clin. Sleep Med..

[5] De Beeck S.O., Dieltjens M., Verbruggen A.E., Vroegop A.V., Wouters K., Hamans E., Willemen M., Verbraecken J., De Backer W.A., Van De Heyning P.H. (2019). Phenotypic Labelling Using Drug-Induced Sleep Endoscopy Improves Patient Selection for Mandibular Advancement Device Outcome: A Prospective Study. J. Clin. Sleep Med..

[6] Epstein L.J., Kristo D., Strollo P.J., Friedman N., Malhotra A., Patil S.P., Ramar K., Rogers R., Schwab R.J., Weaver E.M. (2009). Clinical guideline for the evaluation, management and long-term care of obstructive sleep apnea in adults. J. Clin. Sleep Med..

[7] Arnardóttir E.S., Maislin G., Jackson N., Schwab R.J., Benediktsdottir B., Teff K., Juliusson S., I Pack A., Gislason T. (2012). The role of obesity, different fat compartments and sleep apnea severity in circulating leptin levels: the Icelandic Sleep Apnea Cohort study. Int. J. Obes..

[8] Gabbay I.E., Lavie P. (2011). Age- and gender-related characteristics of obstructive sleep apnea. Sleep Breath..

[9] Jehan S., Zizi F., Pandi-Perumal S.R., Wall S., Auguste E., Myers A.K., Jean-Louis G., McFarlane S.I. (2017). Obstructive sleep apnea and obesity: implications for public health. Sleep Med. Disord. Int. J..

[10] Kastoer C., Benoist L.B.L., Dieltjens M., Torensma B., De Vries L.H., Vonk P.E., Ravesloot M.J.L., De Vries N. (2018). Comparison of upper airway collapse patterns and its clinical significance: drug-induced sleep endoscopy in patients without obstructive sleep apnea, positional and non-positional obstructive sleep apnea. Sleep Breath..

[11] Peppard P.E., Young T., Palta M., Dempsey J., Skatrud J. (2000). Longitudinal study of moderate weight change and sleep-disordered breathing. JAMA.

[12] Segal Y., Malhotra A., Pillar G. (2008). Upper airway length may be associated with the severity of obstructive sleep apnea syndrome. Sleep Breath..

[13] Campos-Rodriguez F., Martínez-García M.A., De La Cruz-Moron I., Almeida-González C.V., Catalán-Serra P., Montserrat J.M. (2012). Cardiovascular mortality in women with obstructive sleep apnea with or without continuous positive airway pressure treatment: a cohort study. Ann. Intern. Med..

[14] Csige I., Ujvárosy D., Szabó Z., Lőrincz I., Paragh G., Harangi M., Somodi S. (2018). The Impact of Obesity on the Cardiovascular System. J. Diabetes Res..

[15] Kalin M.F., Zumoff B. (1990). Sex hormones and coronary disease: a review of the clinical studies. Steroids.

[16] Marin J.M., Carrizo S.J., Vicente E., Agusti A.G.N. (2005). Long-term cardiovascular outcomes in men with obstructive sleep apnoea-hypopnoea with or without treatment with continuous positive airway pressure: an observational study. Lancet.

[17] Price J.F., Fowkes F.G.R. (1997). Risk factors and the sex differential in coronary artery disease. Epidemiology.

[18] Guven S.F., Turkkani M.H., Ciftci B., Çiftçi T.U., Erdogan Y. (2011). The relationship between high-sensitivity C-reactive protein levels and the severity of obstructive sleep apnea. Sleep Breath..

[19] Ryan S., Taylor C.T., McNicholas W.T. (2006). Predictors of Elevated Nuclear Factor-κB–dependent Genes in Obstructive Sleep Apnea Syndrome. Am. J. Respir. Crit. Care Med..

[20] Wainstein M.V., Mossmann M., Araujo G.N., Gonçalves S.C., Gravina G.L., Sangalli M., Veadrigo F., Matte R., Reich R., Costa F.G. (2017). Elevated serum interleukin-6 is predictive of coronary artery disease in intermediate risk overweight patients referred for coronary angiography. Diabetol. Metab. Syndr..

[21] Yokoe T., Minoguchi K., Matsuo H., Oda N., Minoguchi H., Yoshino G., Hirano T., Adachi M. (2003). Elevated levels of C-reactive protein and interleukin-6 in patients with obstructive sleep apnea syndrome are decreased by nasal continuous positive airway pressure. Circulation.

[22] Zen V., Fuchs F.D., Wainstein M.V., Gonçalves S.C., Biavatti K., E Riedner C., Fuchs F.C., Wainstein R.V., Rhoden E.L., Ribeiro J.P. (2012). Neck circumference and central obesity are independent predictors of coronary artery disease in patients undergoing coronary angiography. Am. J. Cardiovasc. Dis..

[23] Wu M.-F., Hsu J.-Y., Huang W.-C., Shen G.-H., Wang J.-M., Wen C.-Y., Huang W.-C. (2014). Should sleep laboratories have their own predictive formulas for continuous positive airway pressure for patients with obstructive sleep apnea syndrome?. J. Chin. Med Assoc..

[24] Iber C., Ancoli-Israel S., Chesson A.L., Quan S. (2007). The AASM Manual for the Scoring of Sleep and Associated Events: Rules, Terminology and Technical Specifications.

[25] (1999). Sleep-related breathing disorders in adults: recommendations for syndrome definition and measurement techniques in clinical research. The Report of an American Academy of Sleep Medicine Task Force. Sleep.

[26] Kazumi T., Kawaguchi A., Sakai K., Hirano T., Yoshino G. (2002). Young men with high-normal blood pressure have lower serum adiponectin, smaller LDL size, and higher elevated heart rate than those with optimal blood pressure. Diabetes Care.

[27] Poirier P., Giles T.D., Bray G.A., Hong Y., Stern J.S., Pi-Sunyer F.X., Eckel R.H. (2006). Obesity and Cardiovascular Disease: Pathophysiology, Evaluation, and Effect of Weight Loss. Circulation.

[28] Deurenberg-Yap M., Schmidt G., A Van Staveren W., Deurenberg P. (2000). The paradox of low body mass index and high body fat percentage among Chinese, Malays and Indians in Singapore. Int. J. Obes..

[29] Deurenberg P., Yap M., A Van Staveren W. (1998). Body mass index and percent body fat: a meta analysis among different ethnic groups. Int. J. Obes..

[30] Arnardottir E.S., Maislin G., Schwab R.J., Staley B., Benediktsdottir B., Ólafsson Í., Juliusson S., Romer M., Gislason T., I Pack A. (2012). The Interaction of Obstructive Sleep Apnea and Obesity on the Inflammatory Markers C-Reactive Protein and Interleukin-6: The Icelandic Sleep Apnea Cohort. Sleep.

[31] Kim J., Lee S.J., Choi K.-M., Lee S.K., Yoon D.W., Shin C., Lee S.G. (2016). Obstructive Sleep Apnea Is Associated with Elevated High Sensitivity C-Reactive Protein Levels Independent of Obesity: Korean Genome and Epidemiology Study. PLoS ONE.

[32] De Ferranti S., Rifai N. (2002). C-reactive protein and cardiovascular disease: a review of risk prediction and interventions. Clin. Chim. Acta.

[33] Khaodhiar L., Ling P.-R., Blackburn G.L., Bistrian B.R. (2004). Serum Levels of Interleukin-6 and C-Reactive Protein Correlate With Body Mass Index Across the Broad Range of Obesity. J. Parenter. Enter. Nutr..

[34] Kern P.A., Ranganathan S., Li C., Wood L., Ranganathan G. (2001). Adipose tissue tumor necrosis factor and interleukin-6 expression in human obesity and insulin resistance. Am. J. Physiol. Metab..

[35] Burke A.P., Tracy R.P., Kolodgie F., Malcom G.T., Zieske A., Kutys R., Pestaner J., Smialek J., Virmani R. (2002). Elevated C-reactive protein values and atherosclerosis in sudden coronary death: association with different pathologies. Circulation.

[36] Din-Dzietham R., Liu Y., Bielo M.-V., Shamsa F. (2007). High Blood Pressure Trends in Children and Adolescents in National Surveys, 1963 to 2002. Circulation.

[37] Haverkate E., Thompson S.G., Pyke S.D., Gallimore J.R. (1997). Mark B Pepys Group Production of C-reactive protein and risk of coronary events in stable and unstable angina. Lancet.

[38] Kleinbongard P., Heusch G., Schulz R. (2010). TNFα in atherosclerosis, myocardial ischemia/reperfusion and heart failure. Pharmacol. Ther..

[39] Lindahl B., Toss H., Siegbahn A., Venge P., Wallentin L. (2000). Markers of Myocardial Damage and Inflammation in Relation to Long-Term Mortality in Unstable Coronary Artery Disease. N. Engl. J. Med..

[40] Van Gaal L.F., Mertens I.L., De Block C.E. (2006). Mechanisms linking obesity with cardiovascular disease. Nature.

[41] Yudkin J.S., Kumari M., Humphries S.E., Mohamed-Ali V. (2000). Inflammation, obesity, stress and coronary heart disease: is interleukin-6 the link?. Atherosclerosis.

[42] Vgontzas A.N., Papanicolaou D.A., Bixler E.O., Kales A., Tyson K., Chrousos G.P. (1997). Elevation of Plasma Cytokines in Disorders of Excessive Daytime Sleepiness: Role of Sleep Disturbance and Obesity. J. Clin. Endocrinol. Metab..

[43] Bettencourt P., Azevedo A., Pimenta J., Friões F., Ferreira S., Ferreira A. (2004). N-Terminal–Pro-Brain Natriuretic Peptide Predicts Outcome After Hospital Discharge in Heart Failure Patients. Circulation.

[44] Ljunggren M., Lindahl B., Theorell-Haglöw J., Lindberg E. (2012). Association between Obstructive Sleep Apnea and Elevated Levels of Type B Natriuretic Peptide in a Community-Based Sample of Women. Sleep.

[45] Maeder M.T., Ammann P., Rickli H., Schoch O.D., Korte W., Hürny C., Myers J., Münzer T. (2007). N-terminal pro-B-type natriuretic peptide and functional capacity in patients with obstructive sleep apnea. Sleep Breath..

[46] Roca G.Q., Redline S., Punjabi N., Claggett B., Ballantyne C.M., Solomon S.D., Shah A.M. (2013). Sleep Apnea Is Associated with Subclinical Myocardial Injury in the Community. The ARIC-SHHS Study. Am. J. Respir. Crit. Care Med..

[47] Randby A., Namtvedt S.K., Einvik G., Hrubos-Strøm H., Hagve T.-A., Somers V.K., Omland T. (2012). Obstructive Sleep Apnea Is Associated with Increased High-Sensitivity Cardiac Troponin T Levels. Chest.

[48] Strehmel R., Valo M., Teupe C. (2016). Natriuretic Peptide and High-Sensitive Troponin T Concentrations Correlate with Effectiveness of Short-Term CPAP in Patients with Obstructive Sleep Apnea and Coronary Artery Disease. Clin. Med. Insights Circ. Respir. Pulm. Med..

[49] Recoquillon S., Pépin J.-L., Vielle B., Andriantsitohaina R., Bironneau V., Chouet-Girard F., Fleury B., Goupil F., Launois S., Martinez M.C. (2018). Effect of mandibular advancement therapy on inflammatory and metabolic biomarkers in patients with severe obstructive sleep apnoea: A randomised controlled trial. Thorax.

